# What athletics bring us? A qualitative research based on John Mo’s thought of “the transfer value of athletics”–evidence from Chinese university graduates

**DOI:** 10.3389/fpsyg.2025.1395298

**Published:** 2025-03-24

**Authors:** Xue Wan, Haohui Liu, Zhihua Yin, Zhen Guo, Bo Liu

**Affiliations:** ^1^College of Physical Education and Health, East China Normal University, Shanghai, China; ^2^High School Attached to Shanghai Normal University (Minhang Campus), Shanghai, China; ^3^Division of Sports Science and Physical Education, Tsinghua University, Beijing, China

**Keywords:** transfer value, athletics, physical education, grounded theory, university sports

## Abstract

As part of the last stage of education, university sports impact all aspects of students’ lives. However, while the value of athletics is not confined to the field, sports researchers have mostly focused on the educational value of athletics, and there is no comprehensive framework for the transfer value of athletics (TVA). As Tsinghua University is considered the flagship of Chinese university sports, this research aims to develop a comprehensive TVA framework by analyzing the memoirs of graduates from Tsinghua in “Tsinghua Sports Influenced My Life” using grounded theory technical approach. In the memoirs, most of the graduates mentioned that the educational value of sports had been transferred into their lives. The findings also demonstrate that the TVA framework consists of five dimensions: competencies, quality, health, personality, and behavior. Analyzing the TVA framework demonstrates that there are three steps for generating TVA: Students participate and enjoy sports, they develop physical literacy, and then transfer the physical literacy into their lives. The memoirs also demonstrate that the formation mechanisms of the TVA include: (1) School sports culture that enables students to establish a positive view of athletics; (2) sports clubs and competition systems that develop students’ physical literacy; and (3) physical educators and legitimate peripheral participation in the community that transfers physical literacy to daily life. This study reveals the value of university sports to students’ lives and provides guidance for universities to develop well-rounded individuals through sports.

## 1 Introduction

“The Transfer Value of Athletics” (TVA) was first developed in a thesis published by Mo (1926), who is considered “the father of modern sports in China” and was the Director of the Physical Education Department of Tsinghua University, the flagship of Chinese sports (Huang, 1986). Tsinghua University honored Mo’s contributions to Chinese sports by naming sports fields and sports classes after him. In the thesis, Mo (1926) proposed that the development of the body, mental character, social adaptation, and morality gained from sports is not confined to the field of sports but will have an impact on the whole life path. Bo Liu, summarized Mo’s TVA theory as follows:

*The core idea of TVA is that sport has a two-fold role, one is to strengthen the body and the other is to educate people, i.e., people are able to translate the good qualities produced in sport into social life*. (Wang and Chen, 2022, p. 3).

In this study, the definition of TVA is based on the views of John Mo and refers to the physical, mental, character, social adaptation, moral, and spiritual development that people acquire in sports. These can be transferred from the field of sports to various aspects of work, study, and life under certain boundary conditions, thus exerting a far-reaching influence on their lives.

Transfer describes the phenomenon of making use of content learned in one setting and applying that to another setting where application may not necessarily be required (Gould and Carson, 2008). Research on the transfer theory in the field of physical education can be divided into two main areas: disciplinary and interdisciplinary. Disciplinary research focuses on the application of transfer theory to the learning of motor skills and is similar to analogical transfer (Barnett and Ceci, 2002). Researchers often find that when individuals develop physical literacy (PL), the sports skills, knowledge, and strategies can be transferred between events. PL is the motivation, confidence, physical ability, knowledge, and understanding needed for individuals to value and participate in physical activity for life (Whitehead, 2007). Individuals with PL are able to actively participate in sports and apply previously acquired knowledge, skills, and strategies to new experiences. First, the learning strategy can be transferred between different sports events. Philip and Kearney (2017) explored the transfer of learning strategies in the learning of other motor skills. Through experimental research, they concluded that students who were taught motor learning strategies were able to successfully recall and use the five-step learning strategy to learn new motor skills within 1 month; moreover, they were more effective than the control group in acquiring motor skills and knowledge. While, the transfer also have the negative effects, it is necessary to careful implementation of skill transfer strategies to maximize positive transfer and minimize negative transfer effects. Second, the power model can be transferred between similar events (Feng et al., 2021). O’Keeffe et al. (2007) applied the transfer of learning theory to the teaching of motor skills and argued that optimizing the sequence of motor skill learning so that learners can benefit from the transfer of prior learning is crucial to the teaching of motor skills. They also investigated the application of transfer in physical education through experiments and explored the effects of two instructional interventions on the learning and performance of basic over-arm throw, badminton overhead strike, and javelin throw (O’Keeffe et al., 2007). In short, they demonstrated that if you are good at badminton, there is a possibility you are good at tennis.

The interdisciplinary research has centered on sport-based youth development, a unique theory that demonstrates that sport can be used as a vehicle to foster psychological, emotional, and life skills development (Holt et al., 2008). For example, teamwork in the context of basketball allows students to notice the importance of team spirit and then use this spirit in daily life. Also, when students take on roles such as coach, referee, or scorekeeper, they will gain personal and social development (Katrijn et al., 2025). This can be summarized as “concrete-abstract-concrete,” representing a high road transfer (Perkins and Salomon, 1988, 1989). Paul Wright et al. (2019) demonstrated that physical education can effectively transfer positive values, attitudes, and behaviors in different contexts and developed the Transfer of Responsibility Questionnaire to assess the transfer process of students in physical education.

Furthermore, the Personal and Social Responsibility Model (PSRM) was developed by Hellison (2011) to explore the transfer process in physical education and other disciplines in relation to the personal and social responsibility developed by students in physical education. Barrie and Stephanie (2015) also demonstrated that the success of facilitating learning transfer in teaching based on the PSRM remains uncertain and that learning transfer is not limited to the field of physical education in general. Manzano Sánchez et al. (2021) explored the implementation of the PSRM in a competitive environment and concluded that, when the PSRM is flexibly adapted and implemented in a competitive youth sports setting, it improves students’ personal and social responsibility, pro-social behavior, and self-efficacy. Based on the research mentioned above, Toivonen et al. (2021) pointed out that although prior studies have demonstrated that the use of Hellison’s (2011) PSRM in physical activities increases participants’ positive values, autonomy, life skills, and pro-social behaviors and positively affects youth development, a lack of qualitative research on the effective content of these programs and their implementation and evaluation persists (Jacobs and Wright, 2018).

The current research demonstrates that many studies have confirmed the value of physical education. However, most of the studies focus on only one aspect of the transfer, such as personal and social responsibility or life skills, but few studies have explored the specific aspects of the TVA. And there is a lack of qualitative research based on prior studies. However, the use of qualitative research methods is necessary. Through empirical methods, qualitative research can convert the more macro viewpoints put forward by John Mo into concrete theories, and use facts to verify and develop his viewpoints, so that people can be more convinced and more accurately recognize the value of university sports for individual development. The findings derived from this study can provide a theoretical basis for the development of university sports, guide universities with different backgrounds to further explore their unique physical education values on this basis, and help universities of all kinds to form their own campus sports characteristics.

## 2 Materials and methods

Grounded theory is a representative qualitative research method in social science. Originally proposed by Glaser and Strauss (1967), grounded theory generally does not make theoretical presuppositions before the beginning of the study and often starts directly from the actual observation, generalizes, and summarizes the experience in the primary data, and then gradually forms a systematic theory. The methodology is well suited to the study of this issue, as we need to condense experiences and formulate theories from the currently available cases from the bottom up. The methodology is well suited to meet the needs of this study. Grounded theory technical approach includes three levels of coding: open coding, axial coding, and selective coding. Through these, the grounded theory can ultimately form a four-tiered framework consisting of the core category, the subsidiary category, the sub-category, and the concept. Based on grounded theory, the specific research process of this study is as follows.

### 2.1 Data collection

The research data of this study is selected from articles in the column “Tsinghua Sports Influenced My Life” on the social media platform “Tsinghua Alumni Association.” The articles in this column are from the memoirs of Tsinghua University alumni for the 110th anniversary of the founding of the university, totaling 110 articles. The individuals selected for this study have included all types of people involved in sports, such as athletes from campus sports teams, general sports enthusiasts, and so on. Also, they are all highly passionate about sports and have maintained good sports habits for many years after graduation. By analyzing all the articles, 14 articles that were not related to the content of this study were excluded. The final total number of materials used for the study was 96 articles.

Three reasons influence the selection of the above material for research. First, the material describes the specific impact of Tsinghua sports on the students’ families, work, study, and other aspects after graduation, which fits the main purpose of this study. Second, Tsinghua University has a good campus sports culture that emphasizes both physical fitness and personality and is one of the most famous sports universities in China because it “educates people through sport.” Tsinghua University’s sports have cultivated a large number of alumni with high morals, strong personalities, good physical fitness, and positive abilities; thus, it is reasonable, feasible, and scientific to use the alumni’s memoirs of the university as the research material. Third, Mo put his TVA philosophy of education into practice during his work in physical education at Tsinghua University, which impacted many students who submitted to “Tsinghua Sports Influenced My Life.” This is an effective starting point for verifying the effect of the transfer value of university sports empirically. To further enhance the reliability of the articles, we also conducted informal interviews with some of the contributors about their growth in participation in sports at Tsinghua University to verify the authenticity of their contributions.

### 2.2 Data analysis

**Figure 1:**
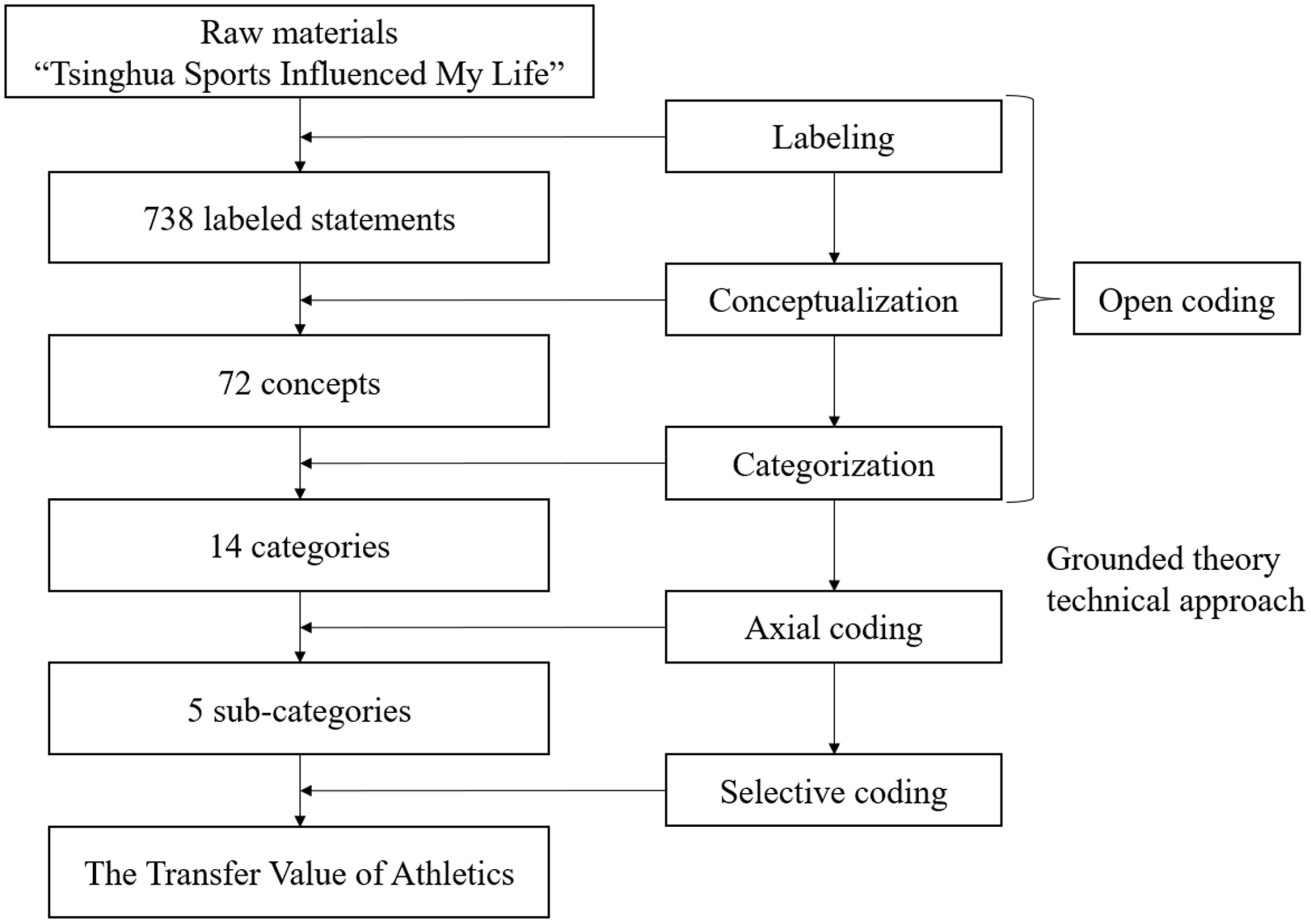
The down-top coding form of grounded theory technical approach.

This study used NVivo to code and analyze the research data. After importing all the research data into the software, the data were coded according to the down-top coding form of grounded theory technical approach (Figure 1).

#### 2.2.1 Step 1: open coding

Open coding is a preliminary analysis of the original research data, which contains three specific parts.

##### 2.2.1.1 Labeling

This study focuses on analyzing the impact of the individual’s college athletic experience; therefore, the impact of sports on individuals was used as the basis of the coding process. The coding process centered on the following questions: (1) Does the author’s statement directly or indirectly reflect the impact of Tsinghua sports? (i.e., was TVA realized by the author?); and (2) How did the author’s statement reflect the influence of Tsinghua Athletics? (i.e., the actual effect of the university’s TVA).

In summarizing the tagged statements for the university-coded logins, the content meaning of the original source material was maintained as much as possible, and this specific process is illustrated in Table 1.

**Table 1:** Example of raw data labeling process.

Type	Raw data content	Open coding: tagging
Data source: “Tsinghua Sports Influenced My Life” alumni’s written remarks Data number: 01	Since I left Tsinghua University nearly 30 years ago, sport has always been an important part of my life, which not only gives me a healthy body, but also makes my life colorful. Sport has been integrated into the blood of Tsinghua students, making us unique. It does not leave us after graduation but is reflected in our daily life and work when we leave Tsinghua University. In the representative team, we learned to unite and love to work hard for collective honor; in the workplace, we know the importance of team combat, encounter difficulties, dare to challenge, and actively face and love the sports atmosphere of the next generation. My two sons are sports lovers; the eldest has been practicing tennis since childhood and was the winner of the “Zheng Jie Cup” youth tennis match. My second son is also the MVP of the high school basketball team. Watching their games is even more intense than my own games. Sports is the best bond of emotional connection in my family.	E1 Bring me a healthy body E2 In the representative team, we learn to be united and love each other E3 We learned to work hard for our collective honor E4 At work, we learn the importance of teamwork E5 Dare to challenge when encountering difficulties E6 Face difficulties positively E7 Brings the love of sports to the next generation

##### 2.2.1.2 Conceptualization

The purpose of open coding is to define the phenomenon, concepts, and categories. In the preliminary conceptualization of the phenomenon, according to the principles of science, the similarity, relevance, the label statement for subdividing and summarizing, level-by-level reduction, and the formation of concepts are included. In the process of concept formation, it is necessary to constantly compare the concepts and merge the synonymous ones with a large degree of similarity to ensure relative independence between the conceptual connotations. After the conceptualization process, 72 concepts were condensed from 738 labeled statements, which were presented using the naming scheme of “DN + concept name” (e.g., D5 environmental adaptive capacity). The details of the process from raw data to conceptualization are illustrated in Table 2.

**Table 2:** Example of a process from source material to conceptualization.

Open coding-conceptualization	Open coding-tagging	Sample source data
D5 Social adaptability	E184 Ability to adapt quickly to a variety of positions	At that time, I really did not know that physical training with strong physical exercise and the formation of high-density rest and rest habits would play a very good role in my later postgraduate study and later foreign exchange and overseas publicity work, which would benefit me for a lifetime. When confronted with various work tasks, active and passive job switching, and balancing the time and energy of teaching, scientific research, and management work, I was able to quickly adapt and quickly grasp the position.
	E614 Quickly adapted to such a tough environment	The dormitories in the factory were very simple, with large bunks (earth beds without heating) like those in the countryside. The windows were still exposed with large cracks. When the north wind blew in winter, the room would freeze. Because of the training of the Tsinghua Motor team, I quickly adapted to such a difficult environment.

##### 2.2.1.3 Categorization

After defining the phenomenon and concept, the next step is to analyze, classify, compare, and summarize the concepts’ meaning and their internal connections; extract the categories from the conceptual clusters; and name the categories. In the process of categorization, it is still necessary to constantly compare and condense the concepts to ensure relative independence between categories. After repeated labeling, conceptualization, and categorization of the research data, the final analysis resulted in 14 categories, which were presented in the form of “CN + category name” (e.g., C2 social adaptability). The process and results of the categorization are illustrated in Table 3.

**Table 3:** Example of categorization process for primary data.

Open coding -categorization	Open coding -conceptualization	Open coding-tagging	Sample source
C2 Social adaptability	D4 Expressive communication ability	E722 Exercised expressiveness E344 Giving the impression of being articulate E591 Became talkative E345 Impressed as an easy communicator	The training of the shooting team has not only trained my skills, but also honed my abilities in other aspects. For example, oral skills and the ability to express myself.
	D5 Social interaction competence	E284 The social skills I developed in the sports team enabled me to integrate quickly with my colleagues E282 Improved our social skills E402 Facilitated social interactions with people E599 It has had a profound effect on the way I interacted with people in my later working life E669 Developed my ability to deal with a wide range of people	In the big family of national sports teams, each team should do a good job of logistical support for each other during college competitions. These social activities have greatly exercised and improved our social skills.

#### 2.2.2 Step 2: axial coding

Axial coding is the second step of coding in the grounded theory method, which refers to the process of linking the categories derived from open coding through the paradigmatic model of “causality → phenomenon → situation → mediating conditions → action/interaction strategies → outcome” based on the completion of the first level of coding. In the process of axial coding, the relationships between different categories need to be analyzed and compared to further explore the main categories. A total of five main categories were summarized and presented in the form of “BN + category name,” including B1 Competencies, B2 Quality, B3 Health, B4 Personality, and B5 Behavior (Table 4).

**Table 4:** Result of axial coding.

Subsidiary category	Category
B1 Competencies	C1 Sports competencies
	C2 Social adaptability
	C3 Working ability
B2 Quality	C4 Morality
	C5 Character
	C6 Spirituality
B3 Health	C7 Physical health
	C8 Mental health
B4 Personality	C9 Disposition
	C10 Interests and hobbies
B5 Behavior	C11 Spread Sports culture
	C12 Family physical education
	C13 Healthy behaviors
	C14 Sports perspectives

#### 2.2.3 Step 3: selective coding

Selective coding is a process through which core categories are selected and involves progressively establishing links between the core categories and other categories, verifying their relationships, and completing the conceptualization of categories that have not yet been fully developed. The data analysis in the selective coding was similar to that of the principal axes coding but at a more abstract level of analysis. Analyzing C1–C14, especially the in-depth analysis of the five main categories of B1–B5, and through comparison, interaction, and questioning in conjunction with the coded primary source records, demonstrated that the core category that united all the other categories was the TVA.

### 2.3 Data testing

After constructing the TVA framework, it was also necessary to conduct saturation and consistency tests on the results. The saturation test helped ensure that the researcher could not obtain additional information to form new categories. Subsequently, 80% of the research data was first used as a sample (total of 76 coded originals), and then the remaining 20% of the sample (total of 20 coded originals) and other relevant data were used for the theoretical saturation test. The results demonstrated that no new nodes appeared (no new categories or relationships were found in the remaining material.), and the coding content could be saturated. At this point, the compilation process was completed; therefore, the TVA framework was more objective and scientific.

The purpose of the consistency test is to avoid errors caused by subjective factors in qualitative research. To ensure the objectivity of the overall coding, the researcher usually needs to test the credibility of the grounded theory to ensure that the results of the grounded theory research are highly reliable. First, 20 paragraphs were randomly selected from all the research samples for the coding test. Second, the author invited two research collaborators to conduct back-to-back coding on the samples. Both collaborators have good qualitative research literacy, have received qualitative research training, and are familiar with NVIVO 11.0 qualitative research coding software.

Finally, after the two co-researchers completed the coding, the author calculated the coding repetition rate for the coding results. The formula for calculation was: consistency factor = number of passages rated consistent/total number of passages. The result for the consistency coefficient of co-researcher A was 19 paragraphs rated as consistent/20 total paragraphs = 0.95. The result for the consistency coefficient of co-researcher B was 18 paragraphs rated as consistent/20 total paragraphs = 0.90. The coding consistency coefficients of the two co-researchers were both ≥0.90, and the results of the checking demonstrated that the researchers’ interpretations were not affected by personal subjective bias and that the research solution had a good degree of reliability.

## 3 Results

**Figure 2:**
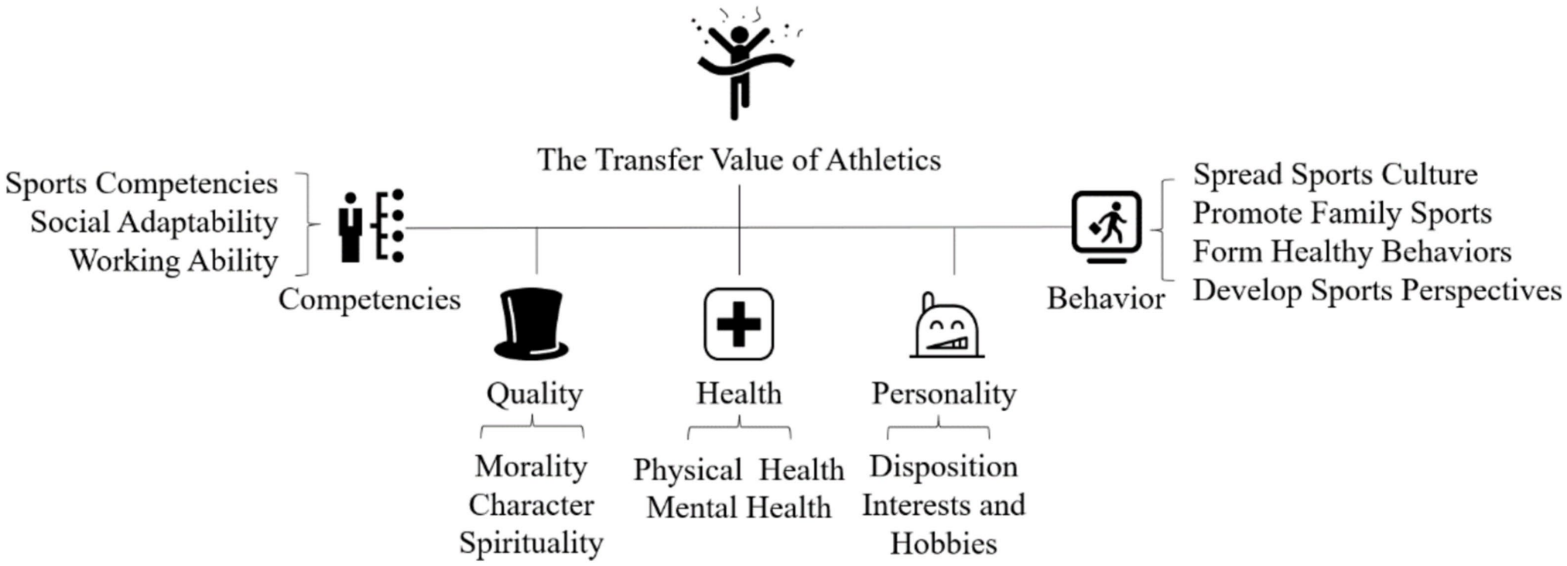
The TVA framework.

Eventually, through the advancement of labeling→ conceptualization→categories→subsidiary categories→core categories, a TVA framework emerged. The framework contains one core category, five sub-categories, 14 categories, and 72 concepts (Figure 2).

### 3.1 Subsidiary category 1: developing comprehensive competencies

The dimension of competencies contains three aspects: sports competencies, social adaptability, and working ability. Among them, sports competencies refer to the ability to become familiar with physical exercise, understand the techniques and tactics, and master basic sports knowledge through practice. Social adaptability specifically includes communication with others, environmental adaptation, social interaction, and cooperation. Work integration ability mainly points to the ability to solve complex problems in the real world, such as learning new knowledge, analyzing problems, and relieving pressure.

### 3.2 Subsidiary category 2: forming good quality

This dimension covers morality, character, and spirituality. Morality generally refers to virtues such as honesty, trustworthiness, fairness, and justice. Character generally identifies the overall style of people in the process of doing things, such as responsibility and tolerance. Spirit includes people’s emotions, will, and other vital signs and general psychological states, such as perseverance, positivity, and enterprising skills.

### 3.3 Subsidiary category 3: promoting physical and mental health

By analyzing the material, the results demonstrate that sports can create health benefits to the participants both physically and mentally, which has been demonstrated by countless studies.

### 3.4 Subsidiary category 4: shaping positive personality

This dimension mainly refers to qualities such as bravery, tenacity, self-confidence, and optimism that participants develop during sports, as well as the formation of sports interests and hobbies that last a lifetime. In the materials, most of the participants mentioned that their university sports experiences enabled them to develop a lifelong passion for participating in sports and helped form positive and stable character, which helped them in their work and life.

### 3.5 Subsidiary category 5: influencing the daily behavior

This dimension mainly covers spreading sports culture, creating a family sports atmosphere, forming healthy behaviors, and developing a positive understanding of sports. Spreading sports culture refers to the ability to actively motivate others to participate in sports and publicize and promote physical exercise. Creating a family sports atmosphere mainly means being able to motivate children and parents to engage in physical exercise and cultivate the exercise habits of their families. Healthy behaviors include developing scientific exercise habits, socializing in sports, and regulating psychology through sports. Forming a positive view of sports is reflected in the ability to understand the educational value of sports.

Overall, as demonstrated in the results of this study, the TVA can improve comprehensive competencies, inculcate good quality, promote health levels, shape positive personalities, cultivate positive behaviors, and produce positive transfers in many aspects of a sportsperson’s lifetime.

## 4 Discussion

The results of this study demonstrate the benefit of the TVA proposed by Mo; that is, the physical, mental, character, social adaptation, moral, spiritual, and other benefits gained by the individual through sport can be transferred and have a positive effect on the life of the individual. In this section, we analyze the TVA’s composition, internal formation mechanism, and future research directions.

The TVA is mainly composed of ability, character, health, personality, and behavior, which correspond to several aspects of PL. Many countries have defined PL and use it as the main goal of physical education. In the United States, PL is categorized into motor skills and physical abilities, sports strategies and awareness, health knowledge and skills, sports behaviors and social interactions, and sports cognition and affective attitudes (Aspen Institute, 2015). In Canada, PL is categorized as affective, physical, cognitive, and behavioral (Longmuir et al., 2015); in Australia, it is physical, mental, social, and cognitive (Keegan et al., 2019); in New Zealand, it is physical, social and emotional, cognitive, and mental; and the International Physical Literacy Association categorizes PL as physical, emotional, and cognitive (Green et al., 2018). Most of the abilities, virtues, health benefits, characteristics, and behaviors involved in this study’s results fit with these PL categories and have the following points in common: first, they demonstrate the combination of physical and mental characteristics, and there is a close connection between the mind and the body. For instance, Participant 1 mentioned her experience with the volleyball team and her narrative reflects that the TVA does not only have an isolated facilitating effect on the body or the mind. Second, the two are interconnected and interact with each other, and the effects of sports on the body, health, and behavior are accompanied by psychological changes:

*The Tsinghua women’s volleyball experience not only strengthened my body and cultivated my tactical thinking, but more importantly, it taught me the spirit of unity and cooperation, the style of hard work and endurance, the indomitable will, and the mentality of not being proud of a victory or discouraged by a defeat. All these have contributed to my academic and career development in a subtle way. Everyone who understands Tsinghua’s sportsmanship understands how physical exercise promotes the body and mind. (Participant 1).*

Third, the TVA reflects the embodied attribute of sport and physical activity as always being the center of the transfer value and the formation of PL. Participant 2 mentioned their embodied experience in the sports team, demonstrating that only when we are physically involved in sports can we realize and gain the great benefits of athletics (Hills, 2007), and it is difficult to form a correct perception of sports just by watching sports activities. Additionally, the TVA only occurs when students overcome the knowledge-doing gap (Wilson, 2007):

*As a fan of the Columbia University fencing team, this season, I have also experienced the various NCAA competition scenes, inter-collegiate interactions, mutual motivation within the team, and have personally felt the sportsmanship spirit in every little bit. I think that this rich experience of having such a very close-knit, mutually reinforcing team together for four years of college will be an unforgettable time for these student-athletes, and if there’s one activity that is the best part of going to college, it’s being on a sports team. (Participant 2).*

Finally, the TVA is often reflected in the form of comprehensive development. Most participants mentioned that their improvement involved four or five dimensions in the results of this study, which reveals that the five TVA dimensions are closely related rather than separated. This feature reflects that sports activities are not only able to cultivate PL but also able to break through the boundaries of disciplines and cultivate the literacy people need in social life, pointing to higher-order interdisciplinary literacy, and supra-disciplinary literacy.

The TVA framework demonstrates that three steps generate the TVA: (1) Students participate and love sports; (2) then, they develop PL; and (3) lastly, they transfer PL into real life. Specifically speaking, physical literacy developed in sports can influence individuals at the psychological and sociological levels. In psychological level, Physical literacy fosters self-efficacy (Zimmerman, 2000) as individuals master skills like swimming or cycling, reinforcing the belief in their ability to tackle new challenges. This confidence spills over into academic or professional settings (e.g., tackling a complex project). Sports require emotional control (e.g., staying calm during a penalty shootout), enhancing emotional intelligence applicable to relationships or decision-making. In sociological levels, social identity theory explains how group membership (e.g., a soccer team) strengthens self-concept, fostering empathy and prosocial behavior in broader contexts (Gutiérrez et al., 2024). According to this theory, team sports cultivate essential skills such as collaboration, communication, and conflict resolution—skills that are directly applicable to workplace group projects, for example, in resolving tactical disagreements.

However, we still do not know how to make students participate, enjoy PE, and develop PL in school settings. Subsequently, engaging students in sports is a prerequisite for the TVA. Dwyer et al. (2006) reported that 97.2% of schools offered school sports programs, yet only 15% of the school student population participated. Furthermore, negative experiences in sports, such as being disliked for being a rookie, might drive students away from sports and, as a result, not cultivate PL.

Specific points that emerged from this study demonstrate the solution to these problems, including developing positive school sports culture, sports club, competition system, and the guidance of physical education teachers.

First, the school sports culture is a prerequisite for the formation of the TVA. According to Peterson and Deal (1998), school culture is the underground stream of values, beliefs, and traditions that build up and influence the daily behavior and actions of everyone at the school and in the school context. Tsinghua University, known as one of the top universities in China, has a strong tradition of campus sports culture and insists on integrating sports into the goal of educating people, which can be reflected in the slogans about sports at different times. Some of the more famous ones are “Sports cannot be qualified to graduate and go abroad,” “Sports is the most important and effective method of education,” “Physical fitness and personality should be emphasized together,” and “No sports, no Tsinghua.” As stated by Participants 3 and 4, under the leadership of the school sports philosophy demonstrated by its slogan, students can form a positive opinion of sports, be motivated to participate in sports, and develop lifelong participation in sports.

According to Liu et al. (2021), the theoretical connotation of the influence on participants through slogans lies in the importation of values from charisma to the collective unconscious, and the sports slogans and philosophy put forward by John Mo and Nanxiang Jiang in accordance with their charisma influence the dominant groups of the charisma community of students and teachers at Tsinghua. In this way, the slogans and philosophy are imported into the collective consciousness, thus influencing the sporting values of Tsinghua students (Liu et al., 2021):

*To work healthily for the motherland for 50 years is one of the calls that Tsinghua University has left the deepest impression on me. I pledged, and I fought for it. Now, I am retired, but I can still be active on the podium, and by this year, I have worked for exactly 60 years. I am excited, I am touched, and I want to thank Tsinghua University for the comprehensive moral, intellectual, and physical education training, and more importantly, I want to thank Tsinghua’s sports, which have been accompanying my life’s study, life, and work. Tsinghua’s environment and cultural atmosphere have cultivated the students’ love and understanding of sports, and Tsinghua’s sports education and rich extracurricular activities have tempered the physical fitness of the students, will, and perseverance and enabled them to develop good habits of perseverance and hard work. These good habits have accompanied me throughout my life and have supported me in my work for my country for more than 60 years. (Participant 3).*

*“Fight to the finish and never give in!” are the words that Mr. John Mo often used to encourage his students on the training ground, and they are also a powerful force within every Tsinghua sportsperson. In a competitive field, the power of the spirit can sometimes play a magical role, and what seems to be a powerful opponent is often most easily broken through at the spiritual level. Tsinghua students have a strong will quality, unity, no defeat, and they can fight back one point at a time even when they are behind by a big margin. This is the evaluation of Tsinghua’s team by teachers from other schools. Tsinghua Ping Pong has always inherited the spirit of never giving up, no matter who is leading or falling behind, and never giving up until the last point. (Participant 4).*

Second, the school sports club and competition system provide opportunities for students to develop PL (Lauren et al., 2021). They represent real-life situations and encourage students to flexibly participate in distinct social contexts such as socialization, cooperation, and competition (Williams, 2017). Researchers highlighted the role of sports organizations in school sports programs that develop well-rounded people rather than simply skilled individuals (Fraser-Thomas et al., 2005). Furthermore, important life skills can be taught through quality youth sports contexts (Bailey et al., 2009). Life skills are the skills that enable individuals to succeed in different environments (e.g., schools, homes, and communities) (Danish et al., 2004). These skills (e.g., goal setting, emotional control, and work ethic) can be facilitated or developed in sports and are then transferred for use in non-sport settings (Gould and Carson, 2008; Jacobs and Wright, 2018). Therefore, providing a field for students to engage in athletics is required.

In the early years, Tsinghua University set up various sports teams, such as volleyball, track and field, soccer, tennis, and basketball teams. This expanded to include sprinting, middle and long-distance running, gymnastics, and other individual sports associations, and Tsinghua University currently has 34 sports clubs. In terms of sports competitions, as Participants 5 and 6 said, most of the alumni mentioned their experiences of participating in “sports activities and competitions,” which included university-level competitions, such as departmental sports day, university sports day, first-year sports day, various kinds of long-distance running activities on campus, and inter-university competitions, such as the Beijing University Sports Day. The participants included both high-level athletes who specialized in sports since childhood and those without any formal training experience:

*I achieved first place in the men’s 400 meters in the Freshman Games, but the result was more than 59 seconds, and I was embarrassed to mention it. Before entering the Tsinghua team, I had never participated in formal training, and I had only participated in the school games once during my secondary school years. Finishing first was an accident; I did not expect to be notified to report to the varsity team, and the surprise of being on the block will not be forgotten. (Participant 5).*

*Tsinghua sports have fostered a strong sense of collective honor among Tsinghua people. In 1981, when the track and field team regained the men’s team championship in Beijing, which had been lost for several years, many students gathered in the small square in the middle of Buildings No. 1 and No. 2, calling out to the team members who lived in the fifth floor of Building No. 1. When the team members appeared on the balcony holding the championship trophy, the group was excited and cheered, and at that moment blood surged to the top of my head, and the pain, fatigue, injuries, and illnesses were not worth mentioning, but I only wanted to train harder in the future to repay my classmates with better results … This sense of collective honor, which runs through the whole process of participating in society through work, spurs me to maintain honor at work through my performance. (Participant 6).*

Subsequently, sports clubs and competitions are a “key medium” in the process of forming TVA. However, students practice sportsmanship, hone their willpower, and cultivate a sense of collective honor in sports activities and competitions, which provide “raw materials” that can be transferred to daily life.

Nonetheless, there are some negative effects of TVA that require intervention by physical educators. For example, in soccer, some players may use unethical means in order to achieve victory. And later, they may also tend to use some illegal means to gain benefits in life. Therefore, it requires physical educators to manage the process of students’ sports to stop unethical behaviors and educate them in time. As Mo stated, the TVA cannot be separated from the physical educator. Teachers’ personalities can have an inspiring impact on students’ cognition and play an important role in modeling students’ behavior (Liu and Kuang, 2001). No rules and regulations can replace the role of the teacher’s personality. In the early years of the Tsinghua campus, Mo’s charisma was a profound force influencing Tsinghua students. His noble sentiments, broad-mindedness, and integrity invariably inspired Tsinghua students. Mo had a strictly enforced work and rest schedule for his daily life, alongside good exercise habits. For example, every morning at 6 am, he practiced the taijiquan (or sword). In addition, in the classroom, Mo carried out tensile strength exercises to maintain muscle strength; in the afternoon, during “forced sports” time, students practiced; and before going to bed, Mo would go for a walk. According to Participant 7, in 1958, despite John Mo being 76 years old already, he not only insisted on exercising but also actively participated in sports competitions. The TVA represents the good qualities that teachers “transfer” to the students. The student’s imitation inclination toward teachers enables physical education teachers to model and positively influence students through charismatic teaching, which can plant the seeds of role models in students’ hearts and encourage them to utilize their positive qualities in their future work and life:

*Prof. John Mo was a role model for our athletes and, in today’s popular parlance, an idol in our hearts. As the head coach, as long as we see him, we immediately felt a rush of energy. Just seeing his hair, face with red light, eyes gleaming, glowing, wearing only a single coat and pants in the winter months, and enthusiastically teaching the technical essentials encouraged us to fight upward for the glory of the motherland. (Participant 7).*

In addition, the legitimate peripheral participation (LPP) in the process of sports participation provides an important impetus for the creation of the TVA. Unlike other disciplines, sports have a great deal in common with the collaborative advancement of programs in real societies, which makes it more likely for the literacies developed through participation in sports to be transferred to everyday life. LPP was proposed by Lave et al. (1991), who argued that learning does not take place through the transmission of knowledge or the copying of the work of others but rather through centripetal participation in the context of a learning curriculum (as opposed to a “teaching curriculum”) in the surrounding community. In this context, “legitimacy” refers to the learner’s eligibility to enter the community of practice; “peripherality” refers not only to the peripheral and unimportant position of the novice but also to a dynamic process of becoming a core member; and “participation” refers to the fact that learning occurs only when the learner is involved in the process of practice.

First, from the perspective of the form of sports, the learning of sports skills is often carried out in the form of a team, (i.e., a community of practice), which is the carrier of LPP. In this community, all members have a clear common goal (to win the game), a clear division of labor (different positions and roles in the basketball team), and a shared pool of experience (to interact with each other in the learning process). TVA requires the presence of common elements between the two (Stage, 1991). The student may be quite comfortable with solving the problems on the worksheet (ordinary learning) but not similar questions in an authentic problem-solving context (the desired transfer) (Shiva, 2019). However, in sports, students join the community of practice as beginners; that is, they can go from LPP to core membership, and this growth process enables them to generate high-passage transfer when faced with similar problems in society that require cooperation, leadership, and creativity.

Through this, the abilities developed in the sports context can be transferred to other contexts, thus realizing the TVA. At the practical level, the implementation of TVA requires the concerted efforts of multiple actors. For coaches, coaches not only focus on skill enhancement in training and competition, but also emphasize teamwork, competitive awareness and fighting spirit. These qualities can be transferred to students’ studies and future work, helping them to better face challenges. In addition, coaches should help students develop good moral values by emphasizing sports ethics, such as fair play and respect for opponents. Such moral qualities will be positively transferred in students’ study and life. For physical education teachers, physical education teachers need to incorporate moral education content into their teaching, such as developing students’ awareness of rules and the spirit of cooperation through sports competitions. These educational elements can help students form positive values that can be transferred to other areas. At the same time, physical education teachers can also develop students’ thinking and problem-solving skills by designing challenging physical activities, such as tactical sports (González-Valero et al., 2024). For policy makers, it is first necessary to improve the university physical education system, and ensure the importance of physical education in school education by formulating relevant policies, such as increasing the proportion of physical education courses and guaranteeing funds and venues for physical education teaching. In addition, local policies should be adopted to promote the construction of university sports culture and create a positive sports culture atmosphere, such as organizing internal and external sports events and strengthening cooperation between universities and social sports organizations.

The above discussion analyzed the TVA connotations and mechanisms. However, it is fundamental to note that this study has limitations. First, the information was collected from the memoirs of Tsinghua University students. Experiences from Tsinghua University sports can only provide a reference for other universities, and it is difficult to offer specific enough guidance for other universities, which need to formulate their own sports implementation plans according to their conditions. In order to solve the above problems, other universities should carry out university physical education on the principle of “goal leads to content.” In terms of goals, they should focus on combining universalization and improvement, so that all students have equal opportunities to participate in sports activities, while at the same time improving their mastery of sports skills. In terms of content, universities can carry out the construction of a school sports system based on their own conditions, such as the establishment of sports clubs favored by students, and the formation of a competition system covering multiple sports and multiple levels. Through the above actions to improve the level of university physical education, so that it can be based on their own conditions to provide students to realize TVA. Second, TVA is mainly oriented to the whole of athletics, not specific sports. As we know, the educational value of different sports is different; for example, open motor skills can cultivate the teamwork spirit of students, but closed motor skills are difficult (Zhang et al., 2024). In the future, this question could be explored by conducting interviews with participants in different programs based on the type of sport, or participant observation. In this way, a program-specific TVA theory could be developed to make the theory more mature and comprehensive. Third, the exploration of the TVA may be incomplete owing to a limited sample size, and in the future, according to the development of the times and the changing needs of talents, the TVA framework can be enriched.

## 5 Conclusion

This qualitative analysis of Tsinghua University students’ memoirs of Tsinghua sports using grounded theory was used to form a TVA framework, which involves five subsidiary categories—competencies, character, health, personality, and behavior—and 14 sub-categories—including sports competencies, morality, physical and mental health, bravery, and spreading sports culture. It also includes 72 concepts and 738 statement labels. The TVA mechanisms include school sports culture that enables students to establish a positive view of athletics, sports clubs, and competition systems to develop students’ PL and for physical educators and LPP in the community to demonstrate the implementation of PL in daily life. The findings of this study can provide a theoretical basis for future university physical education research. In particular, the research design can be based on the findings of this study in exploring how to construct university physical education teaching objectives and how to conduct university physical education teaching quality evaluation. At the same time, based on this study, the research paradigm can be utilized in the future to further explore what transfer values can be gained from the participation of students of different ages in physical activities.

## Data Availability

The raw data supporting the conclusions of this article will be made available by the authors, without undue reservation.

## References

[ref1] Aspen Institute. (2015). Physical literacy in the United States: A model, strategic plan, and call to action. Available online at: https://www.aspeninstitute.org/ (Accessed July 15, 2023).

[ref2] Bailey R. Armour K. Kirk D. Jess M. Pickup I. Sandford R. (2009). The educational benefits claimed for physical education and school sport: an academic review. Res. Pap. Educ. 24, 1–27. doi: 10.1080/02671520701809817

[ref3] Barnett S. M. Ceci S. J. (2002). When and where do we apply what we learn? A taxonomy for far transfer. Psychol. Bull. 128, 612–637. doi: 10.1037/0033-2909.128.4.612, PMID: 12081085

[ref4] Barrie G. Stephanie D. (2015). Teaching personal and social responsibility and transfer of learning: opportunities and challenges for teachers and coaches. J. Teach. Phys. Educ. 34, 152–161. doi: 10.1123/jtpe.2013-0184, PMID: 36626690

[ref5] Danish S. J. Forneris K. Hodge A. (2004). Provincial study of opportunities for school-based Physical activity in secondary schools. Journal of adolescent health, I. Heke. Enhancing youth development through Sport. World Leis. J. 46, 38–49. doi: 10.1080/04419057.2004.9674365

[ref6] Dwyer J. J. Allison K. N. LeMoine K. N.. (2006). A provincial study of opportunities for school-based Physical activity in secondary schools. J. Adolesc. Health 39, 80–86. doi: 10.1016/j.jadohealth.2005.10.004, PMID: 16781965

[ref7] Feng X. Li J. Hu S. Zhao Y. Chen L. Wang N. (2021). An approach to the permeation mechanism of learning transfer and teaching strategy in physical education based on complex network. PLoS One 16:e0243906. doi: 10.1371/journal.pone.0243906, PMID: 33406144 PMC7787532

[ref8] Fraser-Thomas J. L. Côté J. Deakin J. (2005). Youth Sport programs: an avenue to Foster positive youth development. Phys. Educ. Sport Pedagogy 10, 19–40. doi: 10.1080/1740898042000334890

[ref9] Glaser B. Strauss A. (1967). The discovery of grounded theory: Strategies of qualitative research. Nurs. Res. 17:364. doi: 10.1097/00006199-196807000-00014

[ref10] González-Valero G. Ubago-Jiménez J. L. Melguizo-Ibáñez E. Fernández-García R. (2024). Application of the teaching games for understanding model to improve decision-making in sport learning: a systematic review and meta-analysis. BMC Psychol. 12:781. doi: 10.1186/s40359-024-02307-2, PMID: 39722041 PMC11670423

[ref11] Gould D. Carson S. (2008). Life skills development through Sport: current status and future directions. Int. Rev. Sport Exerc. Psychol. 1, 58–78. doi: 10.1080/17509840701834573

[ref12] Green N. R. Roberts W. M. Dwayne S.. (2018). Chartingphysical literacy journeys within physical education settings. J. Teach. Phys. Educ. 37, 272–279. doi: 10.1123/jtpe.2018-0129, PMID: 36626690

[ref13] Gutiérrez D. García López L. M. Segovia Y. (2024). Developing social and school facilitators for a positive school transition through a Sport Education and service-learning programme. Phys. Educ. Sport Pedagog. 1016, 1–16. doi: 10.1080/17408989.2024.2319078

[ref14] Hellison D. R. (2011). Teaching personal and social responsibility through physical activity. Champaign, IL: Human Kinetics.

[ref16] Hills L. (2007). Friendship, physicality, and physical education: an exploration of the social and embodied dynamics of girls’ physical education experiences. Sport Educ. Soc. 12, 317–336. doi: 10.1080/13573320701464275

[ref17] Holt N. L. Tink L. N. Mandigo J. L. Fox K. R. (2008). Do youth learn life skills through their involvement in high school sport? A case study. Can. J. Educ. 31, 281–304. doi: 10.2307/20466702

[ref18] Huang Y. F. (1986). John Mo’s sports discourses. Beijing: Tsinghua University Press.

[ref19] Jacobs J. M. Wright P. M. (2018). Transfer of life skills in sport-based youth development programs: a conceptual framework bridging learning to application. Quest 70, 81–99. doi: 10.1080/00336297.2017.1348304

[ref20] Katrijn O. Frans J. P. Leen H. Van Tartwijk J. (2025). Transferring responsibility in primary school physical education: experiences of five teachers participating in a professional development programme. Teach. Teach. Educ. 153:104844. doi: 10.1016/j.tate.2024.104844, PMID: 40041853

[ref21] Keegan R. Barnett L. Dudley D. Richard T. (2019). Defining physical literacy for application in Australia: a modified delphi method. J. Teach. Phys. Educ. 38, 105–118. doi: 10.1123/jtpe.2018-0264

[ref22] Lauren D. S. Douglas L. G. Wyatt U. Humbert M. L. (2021). Improving school sport: teacher-coach and athletic director perspectives and experiences. Sport Soc. 24, 1554–1573. doi: 10.1080/17430437.2020.1755263, PMID: 40036276

[ref23] Lave J. Wenger E. Wenger E. (1991). Situated learning: Legitimate peripheral participation. Cambridge, UK: Cambridge University Press.

[ref24] Liu B. Kuang H. (2001). Revisiting the transfer value of athletics. J. Tsinghua Univ. 15, 126–130. doi: 10.15877/j.cnki.nsic.20210909.001

[ref25] Liu B. Zhang B. Guo Z.. (2021). Genealogy and deconstruction of university sports slogans and concepts-taking Tsinghua University as an example. Res. Phy. Educ. 35, 1–8.

[ref26] Longmuir P. E. Boyer C. Lloyd M. Yang Y. Boiarskaia E. Zhu W.. (2015). The Canadian assessment of physical literacy: methods for children in grades 4 to 6 (8 to 12 years). BMC Public Health 15, 767–778. doi: 10.1186/s12889-015-2106-6, PMID: 26260572 PMC4532252

[ref27] Manzano Sánchez D. González Víllora S. Valero Valenzuela A. (2021). Application of the teaching personal and social responsibility model in the secondary Education curriculum: implications in psychological and contextual variables in students. Int. J. Environ. Res. Public Health 18:3047. doi: 10.3390/ijerph18063047 33809563 PMC8001341

[ref28] Mo J. (1926). The transfer value of athletics. Springfield: International Young Man’s Christian Associat College.

[ref29] O’keeffe S. Harrison A. Smyth P. (2007). Transfer or specificity? An applied investigation into the relationship between fundamental overarm throwing and related sport skills. Phys. Educ. Sport Pedagogy 12, 89–102. doi: 10.1080/17408980701281995, PMID: 40036276

[ref30] Paul Wright K. Andrew R. Richards J. M.. (2019). Hemphill. Measuring perceived transfer of responsibility learning from Physical Education: initial validation of the transfer of responsibility questionnaire. J. Teach. Phys. Educ. 38, 316–327. doi: 10.1123/jtpe.2018-0246, PMID: 36626690

[ref31] Perkins D. N. Salomon G. (1988). Teaching for transfer. Educ. Leadersh. 46, 22–32.

[ref32] Perkins D. N. Salomon G. (1989). Are cognitive skills context-bound? Educ. Res. 18, 16–25. doi: 10.3102/0013189X018001016

[ref33] Peterson K. Deal T. (1998). How leaders influence the culture of schools. Educ. Leadersh. 56, 2–30.

[ref34] Philip E. Kearney P. J. (2017). Successful transfer of a motor learning strategy to a novel Sport. Percept. Mot. Skills 124, 1009–1021. doi: 10.1177/0031512517719189, PMID: 28685651

[ref35] Shiva H. (2019). Transfer of learning and teaching: a review of transfer theories and effective instructional practices. IAFOR J. Educ. 7, 93–111. doi: 10.22492/ije.7.1.06

[ref36] Stage F. K. (1991). Common elements of theory - a framework for college student development. J. Coll. Stud. Dev. 32, 56–61.

[ref37] Toivonen H. M. Wright P. M. Hassandra M. Hagger M. S. Hankonen N. Hirvensalo M.. (2021). Training programme for novice physical activity instructors using teaching personal and social responsibility (TPSR) model: a programme development and protocol. Int. J. Sport Exerc. Psychol. 19, 159–178. doi: 10.1080/1612197X.2019.1661268

[ref38] Wang S. Chen Y. Y. (2022). From "Tsinghua model" to the thinking of high-quality development of university sports--interview with prof. Sports Sci. 43, 1–8. doi: 10.13598/j.issn1004-4590.2022.05.001

[ref39] Whitehead M. (2007). Physical literacy: philosophical considerations in relation to developing a sense of self, universality and propositional knowledge. Sport Ethics Philos. 1, 281–298. doi: 10.1080/17511320701676916

[ref40] Williams M. K. (2017). John Dewey in the 21st century. J. Inq. Act. Educ. 9, 91–102.

[ref41] Wilson J. (2007). The transfer of learning: participants’ perspectives of adult Education and training. Ind. Commer. Train. 39:232. doi: 10.1108/00197850710755195, PMID: 35579975

[ref42] Zhang J. Xiao W. Soh K. G. Yao G. Anuar M. A. B. M. Bai X.. (2024). The effect of the Sport Education model in physical education on student learning attitude: a systematic review. BMC Public Health 24:949. doi: 10.1186/s12889-024-18243-0, PMID: 38566018 PMC10986141

[ref43] Zimmerman B. J. (2000). Self-efficacy: an essential motive to learn. Contemp. Educ. Psychol. 25, 82–91. doi: 10.1006/ceps.1999.1016, PMID: 10620383

